# Nano-Pulse Stimulation is a physical modality that can trigger immunogenic tumor cell death

**DOI:** 10.1186/s40425-017-0234-5

**Published:** 2017-04-18

**Authors:** Richard Nuccitelli, Amanda McDaniel, Snjezana Anand, John Cha, Zachary Mallon, Jon Casey Berridge, Darrin Uecker

**Affiliations:** Pulse Biosciences, 849 Mitten Rd., Ste 104, Burlingame, CA 94010 USA

**Keywords:** Nano-pulse stimulation, NPS, Apoptosis, Anthracyclines, Caspase, HMGB1, Calreticulin, Ecto-calreticulin, ATP

## Abstract

**Background:**

We have been developing a non-thermal, drug-free tumor therapy called Nano-Pulse Stimulation (NPS) that delivers ultrashort electric pulses to tumor cells which eliminates the tumor and inhibits secondary tumor growth. We hypothesized that the mechanism for inhibiting secondary tumor growth involves stimulating an adaptive immune response via an immunogenic form of apoptosis, commonly known as immunogenic cell death (ICD). ICD is characterized by the emission of danger-associated molecular patterns (DAMPs) that serve to recruit immune cells to the site of the tumor. Here we present evidence that NPS stimulates both caspase 3/7 activation indicative of apoptosis, as well as the emission of three critical DAMPs: ecto-calreticulin (CRT), ATP and HMGB1.

**Methods:**

After treating three separate cancer cell lines (MCA205, McA-RH7777, Jurkat E6-1) with NPS, cells were incubated at 37 °C. Cell-culture supernatants were collected after three-hours to measure for activated caspases 3/7 and after 24 h to measure CRT, ATP and HMGB1 levels. We measured the changes in caspase-3 activation with Caspase-Glo® by Promega, ecto-CRT with anti-CRT antibody and flow cytometry, ATP by luciferase light generation and HMGB1 by ELISA.

**Results:**

The initiation of apoptosis in cultured cells is greatest at 15 kV/cm and requires 50 A/cm^2^. Reducing this current inhibits cell death. Activated caspase-3 increases 8-fold in Jurkat E6-1 cells and 40% in rat hepatocellular carcinoma and mouse fibrosarcoma cells by 3 h post treatment. This increase is non-linear and peaks at 15–20 J/mL for all field strengths. 10 and 30 kV/cm fields exhibited the lowest response and the 12 and 15 kV/cm fields stimulated the largest amount of caspase activation. We measured the three DAMPs 24 h after treatment. The expression of cell surface CRT increased in an energy-dependent manner in the NPS treated samples. Expression levels reached or exceeded the expression levels in the majority of the anthracycline-treated samples at energies between 25 and 50 J/mL. Similar to the caspase response at 3 h, secreted ATP peaked at 15 J/mL and then rapidly declined at 25 J/mL. HMGB1 release increased as treatment energy increased and reached levels comparable to the anthracycline-treated groups between 10 and 25 J/mL.

**Conclusion:**

Nano-Pulse Stimulation treatment at specific energies was able to trigger the emission of three key DAMPs at levels comparable to Doxorubicin and Mitoxantrone, two known inducers of immunogenic cell death (ICD). Therefore NPS is a physical modality that can trigger immunogenic cell death in tumor cells.

## Background

We have been developing a non-thermal, drug-free tumor treatment therapy that applies ultrashort electric pulses in the nanosecond range called Nano-Pulse Stimulation. These pulses differ from those in the microsecond range typically used for electroporation in that NPS is 1000 times shorter and much larger in amplitude. These two differences result in a very different cellular response. Nanosecond pulses are so fast (100–600 ns) that they can penetrate all cells and organelles in the tumor before internal ions can rearrange to charge the membrane capacitance and screen out the cytoplasmic electric field [1]; In addition this short duration leads to pores that are much smaller than those observed following microsecond range electroporation so they only allow small molecules such as ions to pass through them [2]. These nanopores allow calcium ions to flow into the cytoplasm from both the endoplasmic reticulum (ER) and the extracellular space, causing ER-stress [3, 4] and generating an increase in reactive oxygen species (ROS) [5, 6], and other components in the apoptotic cascade that result in cellular death.

The large amplitude of these pulses (30 kV/cm) means that they can permeabilize both the small organelle membranes as well as the outer plasma membrane. It takes about 500 mV to electroporate each membrane in an organelle so the field across a 1 μm wide organelle must be 1 V per 1 μm which is equivalent to 10 kV/cm. This field strength only delivers fractions of a Joule with nanosecond pulses but would vaporize the cell if applied for microseconds. Therefore, the main characteristic that sets nanosecond pulsed fields apart from the microsecond domain is their ability to electroporate internal membranes. This unique feature first observed by Schoenbach’s group [7] allows nanosecond pulses to be used to tease new responses out of cells such as the initiation of apoptosis and tumor ablation. These characteristics, as well as data demonstrating that treatment of a primary tumor with NPS can inhibit the growth of a secondary tumor [8, 9], indicates a cell death modality that is immunogenic in nature. One form of cell death known to elicit an immune response through ER-stress and ROS production is an immunogenic form of apoptosis [10].

Apoptosis is the programmed cell death pathway used by most of the cells in our bodies when their useful life is over and they need to be replaced. Under normal physiological conditions, apoptosis is tolerogenic and does not mount an immune response [11]. Under conditions of ER-stress the cells undergoing apoptosis may emit signals of distress that are recognizable to the immune system called danger-associated molecular patterns (DAMPs). Normally when our immune system encounters neoantigens resulting from genetic mutations these non-self antigens trigger an immune response that eliminates them. However cancer cells have discovered various techniques that allow them to evade the immune system and form tumors [10, 12]. The immune stimulation that results from the emission of DAMPs causes the immune system to effectively “wake-up” and take notice of abnormal cancer cells, launching an immune response [13, 14].

The phenomenon of ICD was first discovered when specific chemotherapeutics were shown capable of producing an antitumor immune response [15–17] through collateral damage to the ER. A number of treatments that stimulate ER stress through indirect means fall into this category (i.e. anthracyclines, oxalplatin, UV-irradiation etc.) and have been labeled Type I inducers of ICD. Hypericin-photodynamic therapy (Hyp-PDT) and certain oncolytic viruses, cause direct ER-stress, and are considered Type II inducers [18–20]. The commonality amongst each of these agents is their ability to trigger ER stress through either direct (Type II) or indirect (Type I) modalities, stimulating the release of DAMPs [10, 21]. The three DAMPs that have a demonstrated role in the immunogenicity of virtually all ICD inducers are: the translocation of calreticulin (CRT) from the ER to the outer leaflet of the plasma membrane, the secretion of ATP and the release of the high mobility group box 1 (HMGB1) protein that physiologically acts as a DNA chaperone in the nucleus [22].

NPS is a physical method of cell death that directly induces ER stress through the efflux of Ca^2+^ ions. There are also physical effects on other subcellular structures, including the mitochondria [23]. Several other physical modalities have been shown to effectively elicit antitumor immunity through a form of immunogenic cell death. Radiotherapy has been shown to induce many of the DAMPs, and mice “vaccinated” with dendritic cells loaded with irradiated cancer cells are immune to challenge with live syngeneic cells [13, 24]. Other physical treatments with some evidence for stimulating ICD include ultraviolet light, hydrostatic pressure, photodynamic therapy and mild hyperthermia [13].

Here we sought to characterize cell death induced by NPS treatment in three malignant cell lines. We provide evidence that NPS treatment initiates all of the classic DAMPs indicative of ICD and at levels that are comparable with two anthracycline chemotherapeutics that are known ICD inducers, doxorubicin and mitoxantrone [10]. We demonstrate that NPS is a physical modality capable of inducing an immunogenic form of cell death comprised of features of apoptosis when delivered at specific energies.

## Methods

### Cell lines

The MCA205 murine fibrosarcoma cell line was obtained from Andrew Weinberg, Providence Portland Medical Center, Portland, Oregon and cultured in DMEM (containing 4 mM L-glutamine, 4500 mg/L glucose, 1 mM sodium pyruvate and 1500 mg/L sodium bicarbonate), with 10% FBS and 1% Pen/Strep. McA-RH7777 rat hepatocarcinoma cells were obtained from ATCC (CRL-1601) and also cultured in DMEM media. The Jurkat E6-1 human T-cell leukemia cell line was obtained from ATCC (TIB-152) and cultured in RPMI 1640 (containing 2 mM L-glutamine and 25 mM HEPES), with 10% FBS and 1% Pen/Strep.

### Nano-Pulse Stimulation (NPS)

To apply 100 ns pulses we used a pulse-forming network with an electrically-triggered spark gap as a switch as described previously [9]. Briefly, a series of capacitors and inductors are designed as a pulse-forming network to release their stored charge in 100 ns. They are charged up to the desired voltage and the pulse is then triggered electrically to release the stored charge using a spark gap. This spark is very fast and results in a signal rise time of less than 30 ns. The voltage and current applied in all pulses were recorded so that the energy delivered (J/mL) could be easily calculated as the product of the electric field (kV/cm) and current density (A/cm^2^) applied, multiplied by the pulse duration (10^−7^ s) and the pulse number.

### NPS treatment to measure viability and presence of apoptosis

10^6^ cells suspended in 800 μl of media were placed into 4 mm-wide electroporation cuvettes (Cell Projects# EP-104, Kent, UK) for treatments ranging from 6 to 25 kV/cm, and 3.5x10^5^ cells suspended in 200 μl of media were placed into 2 mm electroporation cuvettes (Life Technologies# P45050) for treatments of 30 kV/cm. At a given treatment voltage, cells were treated with the necessary number of 100 ns pulses to meet the total treatment energy requirements ranging from 1.0 to 50 J/mL.

### Viability assay

We used the PrestoBlue® (Invitrogen) Viability Assay to measure cell viability. PrestoBlue is a cell-permeant compound that is blue in color and virtually nonfluorescent. When added to cells, the PrestoBlue® reagent is modified by the reducing environment of the viable cell and turns red in color and becomes highly fluorescent. After treating cells in a cuvette we placed three aliquots containing 80 μl of cells in DMEM on a 96 well plate. The plate was placed in a CO_2_ incubator at 37^o^ C for 2.5 h before 10 μl PrestoBlue® was added to each well. After 15 min, Fluorescence was measured in a SpectraMax i3 plate reader (Molecular Devices; Sunnyvale, CA). Three samples are averaged for each measurement and normalized to the unpulsed cells and positive (fully ablated) control.

### Caspase activation

Activation of combined caspase-3 and caspase-7 was assessed using the Caspase-Glo® 3/7 Assay (Promega). Following NPS treatments, approximately 1.5x10^4^ cells were plated onto three wells each of a 96 well assay plate containing pre-equilibrated media. Previous studies indicated that caspase-3 could be detected by 3 h post NPS treatment [8], so cells were then incubated for 3 h at 37^0^ C, and 5% CO_2_. Caspase-Glo reagent was added to each well at a ratio of 1:1 with cell culture media. This reagent contains proluminescent caspase-3/7 substrate, which contains the tetrapeptide sequence, DEVD. This substrate is cleaved to release amino-luciferin, a substrate of luciferase used in the production of light. Cell lysis results, followed by caspase cleavage of the substrate and generation of luminescence. Samples were incubated for an additional 30 min at room temperature, protected from light, and with gentle agitation. Sample luminescence was then measured using the Molecular Devices Spectramax i3 plate reader. Caspase activation was normalized to untreated samples by dividing the raw luminescence units (RLUs) of pulsed samples by the RLU value of untreated controls.

### Annexin V/7-AAD apoptosis detection assay

The PE Annexin V Apoptosis Detection Kit I (BD Biosciences; San Jose, CA ) was used to assay the percentage of cells undergoing the stages of apoptotic cell death. Cells were treated with NPS (as described above) and then incubated at 37 °C with 5% CO_2_. Cells were harvested at 3- and 24-h post treatment. After harvesting, cells were washed twice with 1X PBS (wash: suspend in 100 μl buffer; centrifuge at 1200 rpm for 5-min at 4 °C) followed by resuspension in 100 μl of Annexin V/7-AAD staining cocktail (1 μl PE Annexin V and 1 μl 7-AAD in 100 μl 1X Annexin Binding Buffer). Cells were protected from light and stained for 15-min at room temperature. After staining, cells were centrifuged at 1200 rpm for 5 min at 4 °C and the staining buffer was removed. Cells were then washed once in 100 μl, 1X Annexin Binding Buffer (see wash conditions above) followed by resuspension in 200 μl 1X Annexin Binding Buffer for flow cytometric analysis.

Stained cells were analyzed on a Beckman Cytoflex flow cytometer. Cells were gated based upon Annexin V binding (PE Annexin V: Ex 488/Em 578) and cell viability (7-AAD: Ex 488/Em 647). Gated cells were binned into 4 populations based upon stage of cell death: live viable cells (PE Annexin V-/7-AAD-); early apoptotic (still viable) (PE Annexin V+/−AAD-); late stage apoptotic/necrotic (non-viable) (PE Annexin V+/7-AAD+); very late stage cell death (non-viable) (PE Annexin V-/7-AAD+). Binned populations were expressed as % of total cells.

### Markers of immunogenic cell death/apoptosis

#### NPS treatment

One million cells were suspended in 800 μl media and placed into a 4 mm electroporation cuvette for each treatment. The pulse parameters were fixed (15 kV/cm, 100 ns, 2 pps) and energy delivery was controlled by varying the pulse number. Cells from each cell line received five total treatments, ranging in energy between 5 and 50 J/mL.

#### Anthracycline treatment

500,000 cells were suspended in 400 μl media containing one of two concentrations of doxorubicin (25 and 100 μM) or mitoxantrone (4 and 10 μM). 500,000 cells from each NPS and each anthracycline treatment group, as well as 500,000 untreated cells were seeded in a 24-well plate and placed into a cell culture incubator (37 °C; 5% CO_2_) for 24 h. Following incubation, plates were centrifuged (1200 rpm for 5 min) and cell culture supernatants were collected to measure levels of ATP and HMGB1. Cells were harvested to measure levels of cell surface calreticulin (ecto-CRT) expression.

### ATP assay

Extracellular ATP was measured using the ATP Determination Kit (Molecular Probes; A22066), a luciferin-based bioluminescent assay. 10 μl of cell culture supernatant or ATP standard was placed into a 96-well white assay plate with 90 μl of a reaction buffer, containing both firefly luciferase and luciferin. Since luciferase requires ATP to produce light when reacting with luciferin, the amount of bioluminescence is directly proportionate to the amount of ATP in the sample. Luminescence of all wavelengths was detected and read using the Molecular Devices SpectraMax I3 plate reader. A standard curve was generated in the accompanying SoftMax Pro software and used to interpolate sample concentrations. However, due to varying concentrations in the untreated samples, we decided to normalize to untreated samples instead of presenting absolute concentration values.

### HMGB1 ELISA

HMGB1 release was measured with the HMGB1 Detection Kit (Chondrex, Inc.; Redmond, WA). This ELISA contained an antibody that cross-reacts with mouse, rat and human epitopes, which enabled us to detect the HMGB1 protein in all three of our treated cell lines. 25 μl cell culture supernatant was diluted in 25 μl of sample/standard dilution buffer for a total sample volume of 50 μl. A volume of 50 μl of each HMGB1 standard and 50 μl of each sample was placed into duplicate wells of a 96-well plate containing both capture and detection antibody. The assay was performed according to the instructions detailed by the manufacturer. After completing the assay, the sample OD values were read at 450 nm using the Molecular Devices SpectraMax i3 plate reader. A standard curve was generated in the SoftMax Pro software and used to interpolate sample concentrations.

### Ecto-Calreticulin (CRT) expression

#### Washing and staining

After harvesting, cells were resuspended in 1 mL cold PBS, centrifuged (1200 rpm for 5 min at 4 °C) to wash, and then supernatant was discarded. After washing twice, the cells were resuspended in 100 μl PBS. 1 μl of Zombie Aqua (ZA) (BioLegend; San Diego, CA), an amine-reactive fluorescent viability dye, was added to each sample and incubated for 20 min in the dark at room temperature. Following the incubation, cells were centrifuged (using above parameters) and the supernatant was discarded. Cells were resuspended in Cell Staining Buffer (BioLegend; San Diego, CA) and washed twice following the same procedures used above. Cells were resuspended in 100 μl diluted (1:75) primary anti-CRT antibody (ThermoFisher; Waltham, MA) and incubated at room temperature for 30 min. After following wash procedures, cells were stained in 100 μl diluted (1:200) Alexa Fluor 647 conjugated secondary antibody (Thermo Fisher; Waltham, MA) and incubated for 30 min at room temperature in the dark. After a final series of washes, cells were resuspended in 200 μl staining buffer.

### Flow cytometry

Cells were analyzed on a Beckman CytoFlex flow cytometer. Cell populations were gated based upon viability (Zombie Aqua: Ex 405 nm/Em 525) and ecto-CRT expression (anti-CRT/Alexa Flour 647: Ex 637 nm/Em 660). Gated cells were binned into four populations: ZA^−^/CRT^+^ (Viable/Expressing CRT); ZA^+^/CRT^+^ (Non-viable/Expressing CRT); ZA^−^/CRT^−^ (Viable/Not expressing CRT); and ZA^+^/CRT^−^ (Non-viable/Not expressing CRT). Binned populations were expressed as % of total cells.

### Varying conductivity to study the current density dependence

Conductivity of the solutions in the electroporation cuvettes was adjusted by mixing iso-osmotic solutions of sucrose and DMEM. We added 1 mM MgCl_2_ and 10 mM Tris–HCl to 280 mM sucrose and adjusted the solution to pH 7.4. We also adjusted the osmolarity to match that of DMEM before mixing the solutions. By combining different proportions of the two solutions we could arrive at a range of current densities from 10 to 200 A/cm^2^ for a constant 20 kV/cm E field.

## Results

### NPS and cell viability

**Fig. 1:**
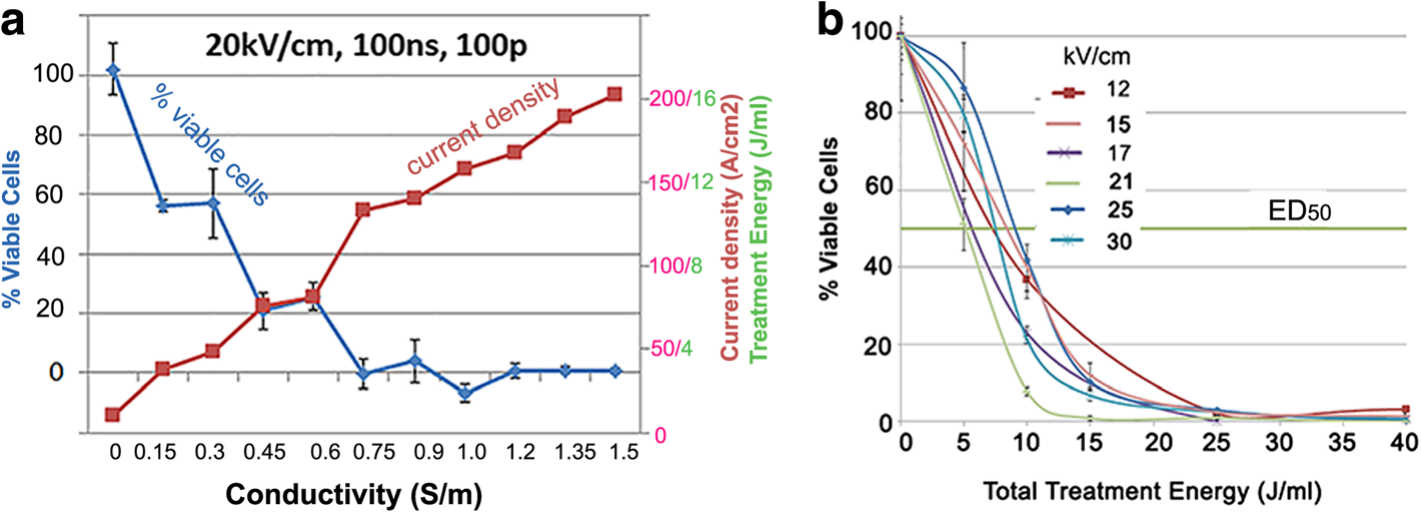
Dependence of McA-RH7777 hepatocellular carcinoma cell viability on current density and treatment energy. **a** With constant pulsed E field parameters of 20 kV/cm and a pulse number of 100, the ablation response is inhibited when less than 50 A/cm^2^ is flowing through the hepatocarcinoma cells; **b**. Percent viability 3.5 h post treatment versus the total treatment energy generates curves that are very similar for most applied E fields, suggesting that it is the energy delivered to the cell rather than the E field that is the more critical parameter for determining ablation efficacy

As a first step in characterizing the cellular response to NPS, we used the McA-RH7777 rat hepatocellular cell line to study the dependence of cell viability on the total treatment energy in vitro using the PrestoBlue® assay, 3.5 h after NPS treatment (Fig. 1). Treatment energy was calculated by multiplying the number of pulses by the product of the applied voltage and current and pulse width. We have previously demonstrated that the nanoelectroablation effect could be observed for field strengths greater than 10 kV/cm [25]. By varying the conductivity of the medium, we have confirmed the initial observation by Silve, et al. [26, 27] that no significant cell death is observed from nanosecond pulsed electric field application when the current density flowing across the cells is below a threshold level. There is very little cell death in response to 100 pulses, 100 ns long and 10–20 kV/cm when only 0 to 20 A/cm^2^ is flowing across the cells. Between 20 and 50 A/cm^2^ the cell death begins to increase in a linear manner (Fig. 1a). Above 50 A/cm^2^, the ablation efficiency increases sharply and it is nearly 100% for current densities greater than 100 A/cm^2^ which corresponds to 8 J/mL (Energy (J/mL) = kV/cm * A/cm^2^ * pulse duration * pulse number). This imposes additional conditions on successful nanoelectroablation. Not only must the field strength exceed 10 kV/cm, but the current density must exceed 50 A/cm^2^. This observation led us to consider the energy delivered to the cell since it depends on both current and field strength.

We plotted percent viable cells versus treatment energy for six different field strengths (Fig. 1b). When energy is held constant the curves practically overlap, demonstrating that field strength is not predictive of the ablative efficacy of NPS. In contrast, varying the energy delivered during NPS is highly predictive of ablative efficacy. The ED_50_ for ablation is around 10–15 J/mL for a wide range of field strengths.

### Apoptosis assays

#### Caspase 3/7 activation

**Fig. 2:**
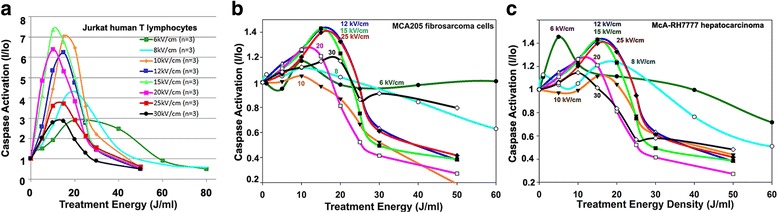
Dependence of caspase 3/7 activation on the treatment energy for three different cell types. The activated caspase levels were measured 3.5 h after applying the indicated energy for the field strength indicated on each curve

We studied the activation of caspase 3/7, 3.5 h after NPS treatment in three malignant cell lines, human Jurkat E6-1 T-lymphocytes, murine MCA205 fibrosarcoma cells and rat McA-RH7777 hepatocarcinoma cells. The strongest response was observed in the human Jurkat cells (Fig. 2a) where the levels of activated caspase 3/7 increased up to nearly 8-fold. The activation of caspase 3/7 in both adherent cell lines (MCA205 and McA-RH7777) only increased to a peak value of about 40% higher than controls (Fig. 2b, c). There was also much more variability in the maximum change in caspase activation from control values noted in the adherent lines.

The analysis of caspase activation by NPS reveals some very interesting characteristics. When caspase activation at various applied field strengths is plotted versus treatment energy, the curves are fairly uniform (Fig. 2). Nearly all of the pulse field strengths applied exhibited caspase activation peaks in the 10–20 J/mL range. This is very close to the 10–15 J/mL range of the cell viability ED_50_ showing a relationship between caspase activation energy and ablative efficacy. For treatment energies greater than 20 J/mL, the activated caspase fell to levels below control values, suggesting the majority of cells succumbed to necrosis at higher energy levels.

One unexpected result revealed in these studies is that the maximum increase in caspase activation was not observed for the highest field strengths tested of 25 and 30 kV/cm. Instead, the peak response was obtained in response to applied fields in the 12–20 kV/cm range and the response was smaller for both lower and higher field strengths (Fig. 2a).

### Annexin V/7-AAD detection

Although caspase-3 activation can indicate the presence of caspase-3-dependent apoptosis, we were interested in the overall percentage of cells that were succumbing to apoptosis verses some other cell death modality. In order to do so we used an Annexin V/7-AAD flow cytometric - based detection assay. PE Annexin V binds phosphatidylserine (PS) which is translocated from the inner to the outer leaflet of the plasma membrane during early apoptosis before loss of membrane integrity, making it a good marker of early apoptosis. In contrast 7-AAD is only capable of crossing the cell membrane and binding to nucleic acids when cells are no longer viable and have become permeable. Detection of Annexin V alone indicates an early stage of apoptotic cell death where the cells are still viable and the membrane is still intact. The presence of both Annexin V and 7-AAD indicates a later stage of cell death where the cells are no-longer viable and membranes have lost their integrity and become permeable.

**Fig. 3:**
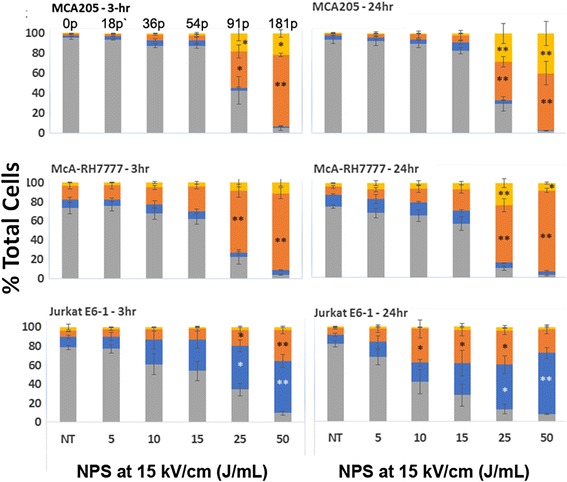
PE Annexin V and 7-AAD labeling to determine stage of apoptotic cell death at 3 h and 24 h. The percent of cells at different stages is indicated by color. *Gray bars* represent live viable cells; *Blue bars* represent cells in the early stages of apoptosis (PE Annexin V+/7’AAD-); *Orange bars* represent cells in the later stages of apoptosis (PE Annexin V+/7’AAD+); *Yellow bars* represent cells in the latest stages of cell death (PE Annexin V-/7-AAD+). The number of pulses applied to achieve the indicated J/ml for all cell lines are indicated above the MCA205 plot. Significant difference from untreated controls by one-way ANOVA with between group analysis performed using the Dunnett’s test. *p < 0.05; **p < 0.01

After NPS treatment, we measured the percent of cells undergoing these various stages of cell death at 3 h (when we measure caspase-3 activation) and again at 24 h (when we measure DAMPS) to follow the progression. The percent of cells undergoing some stage of cellular demise shows an overall increase between 3 and 24 h. Also, the percent of cells that are in the latest stages of cell death clearly increased over time showing progression between 3 and 24 h, particularly at energies above 15 J/mL (Fig. 3).

There appears to be very little cell death in the MCA205 cell line at lower energies (5–15 J/mL). The percent of McA-RH7777 cells that are dead or dying at lower energies is also comparatively small to that seen at energies ≥ 25 J/mL. In both cell lines the percent of cells undergoing cell death increases significantly at 25 J/mL (Fig. 3). In contrast, the Jurkat E6-1 cell line shows a steady increase across energies. Unlike the other two cell lines, there does not appear to be a particular energy threshold at which cells succumb.

Interestingly, the percentage of cells undergoing early apoptosis in the MCA205 and McA-RH7777 cell lines follows a different pattern across energies than do cells undergoing the later stages of cell death. The number of cells in the early stages of apoptosis, when cells are still viable, peaks at 15 J/mL and then drops off at 25 J/mL, showing the opposite pattern from those cells that are in the latest stages of cell death, which significantly increase in percentage at 25 J/mL. The Jurkat E6-1 cell line behavior again contrasts to the other two cell lines. The percent of cells in early apoptosis continues to increase as energy increases, similar in pattern to those at later stages of cell death.

### Ecto-calreticulin expression following NPS and anthracycline treatment

**Fig. 4:**
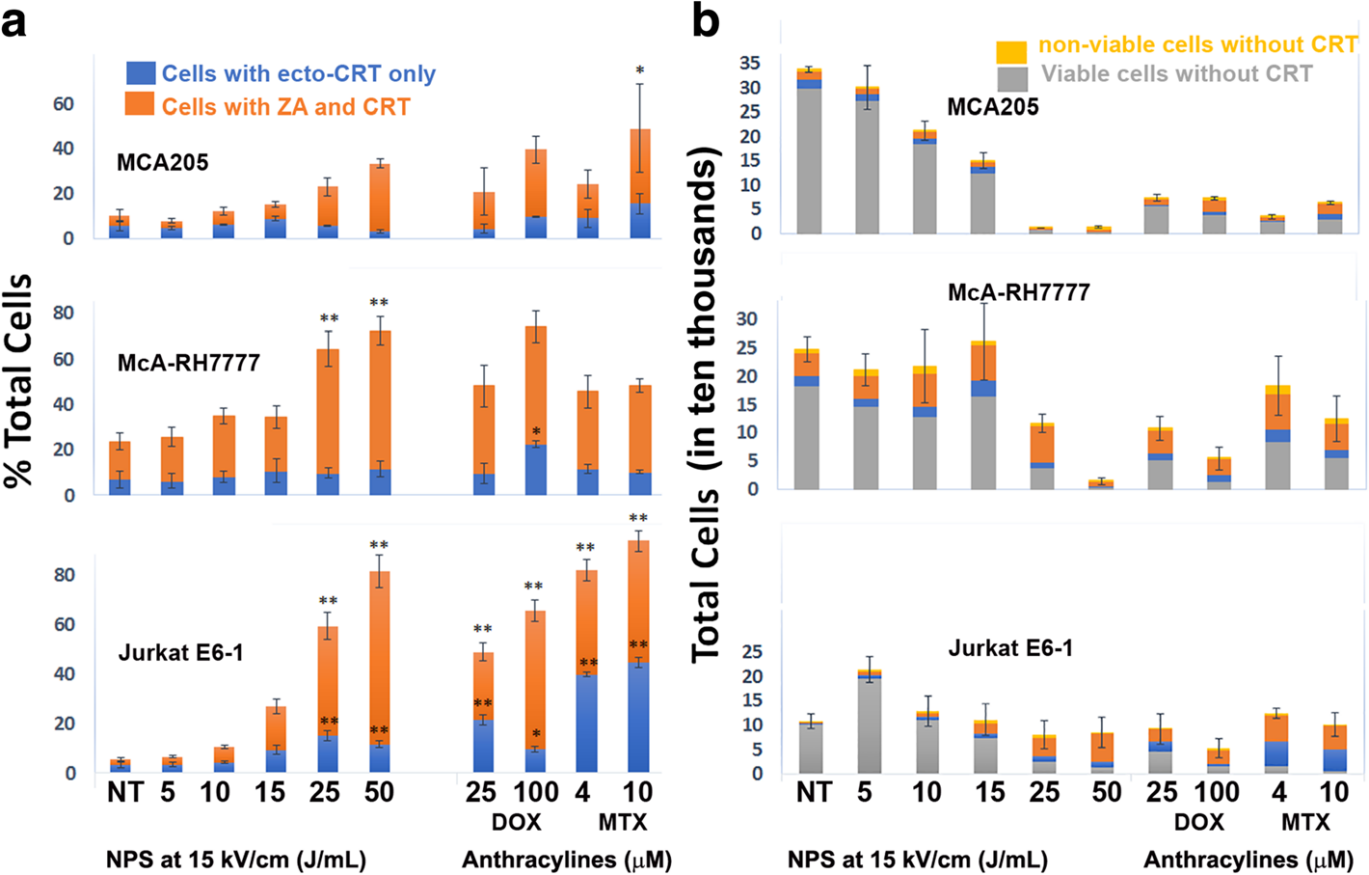
Ecto-calreticulin (CRT) detected by flow cytometry on three different cell lines 24 h after treatment with NPS, doxorubicin (DOX) or mitoxantrone (MTX). **a** Percent of total cells expressing ecto-CRT. *Blue bars* indicate viable cells labeled with CRT that did not label with Zombie Aqua (ZA). *Orange bars* indicate cells that labeled with both ZA and CRT and *yellow* indicates non-viable cells without CRT. Significant difference from untreated controls by one-way ANOVA with between group analysis performed using the Dunnett’s test. *p < 0.05; **p < 0.01. **b** Total cells detected by flow cytometry in four classifications; *Blue* represents those viable cells with ecto-CRT; *grey* indicates viable cells without ecto-CRT; *Orange* indicates non-viable cells with CRT and *yellow* indicates non-viable cells without CRT

We used flow cytometry to measure the percentage of tumor cells expressing cell surface calreticulin (ecto-CRT) after treatment with NPS at a range of energies or with two different anthracyclines (doxorubicin and mitoxantrone) at two different concentrations (Fig. 4a-b). Cell surface binding of anti-CRT was distinguished from internal CRT labeling by selecting cells that did not label with Zombie Aqua, indicating that they would be impermeable to the CRT antibody. The percentage of cells expressing ecto-CRT increased as NPS treatment energy increased for all three cell lines reaching levels comparable to that resulting from anthracycline treatment between 25 and 50 J/mL (Fig. 4a). The percent of non-viable cells with membranes permeable to Zombie Aqua more than doubles after 15 J/mL. Additionally, there is a considerable drop-off in the total number of viable cells at 25 J/mL (Fig. 4b). Of the total cells that remained, more than 50% of the McA-RH7777 and Jurkat E6-1 cells expressed calreticulin on their surfaces at 25 J/mL. The percent of MCA205 cells that expressed calreticulin was lower but followed the same pattern of increase at 25 J/mL. Additional support for this ecto-CRT appearance is found in a previous publication in which we show immunofluorescence images of ecto-CRT binding on the surfaces of NPS-treated McA-RH7777 cells, human pancreatic cancer cells and murine squamous cell carcinoma cells [9].

### ATP secretion after NPS treatment

**Fig. 5:**
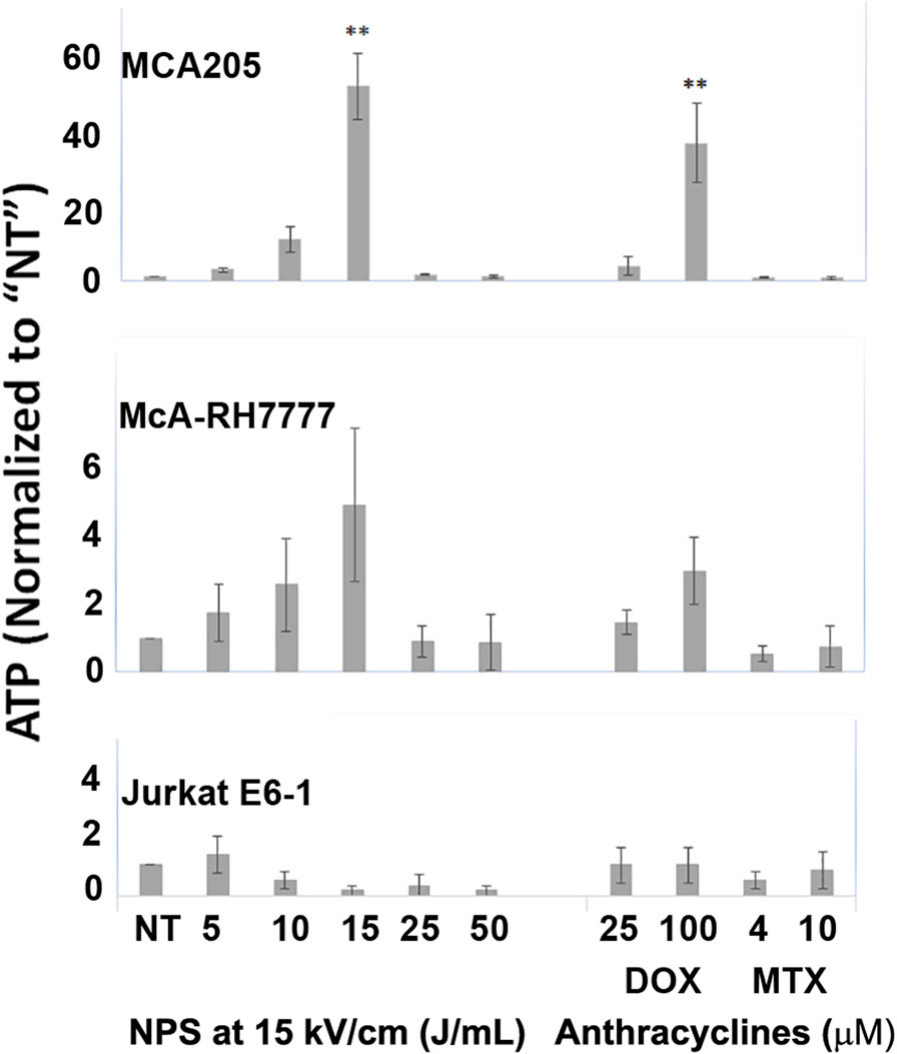
ATP released by three cell lines 24 h after treatment with either NPS, or DOX or MTX. All measurements were normalized to the untreated levels of ATP. Significant difference from untreated controls by one-way ANOVA with between group analysis performed using the Dunnett’s test. *p < 0.05; **p < 0.01

The ATP released from both MCA205 and McA-RH7777 cells 24 h after NPS treatment showed a well-defined peak at 15 J/mL (54 pulses;15 kV/cm) with a sharp decline at 25 J/mL (Fig. 5). The ATP release was highest at 15 J/mL in both cells lines and significantly so in the MCA205 compared with untreated cells. Cells treated with the higher concentration of doxorubicin (100 μM) released the second highest amount of ATP and the levels were also significantly higher than untreated cells in the MCA205 cell line. The mitoxantrone-treated cells released a comparatively small amount ATP at both high and low concentrations (4 and 10 μM).

Jurkat E6-1 ATP secretion levels were much lower than those observed from the adherent cell lines. ATP levels measured in the NPS or anthracycline treatment groups were not significantly different from background for any condition.

### HMGB1 after NPS treatment

**Fig. 6:**
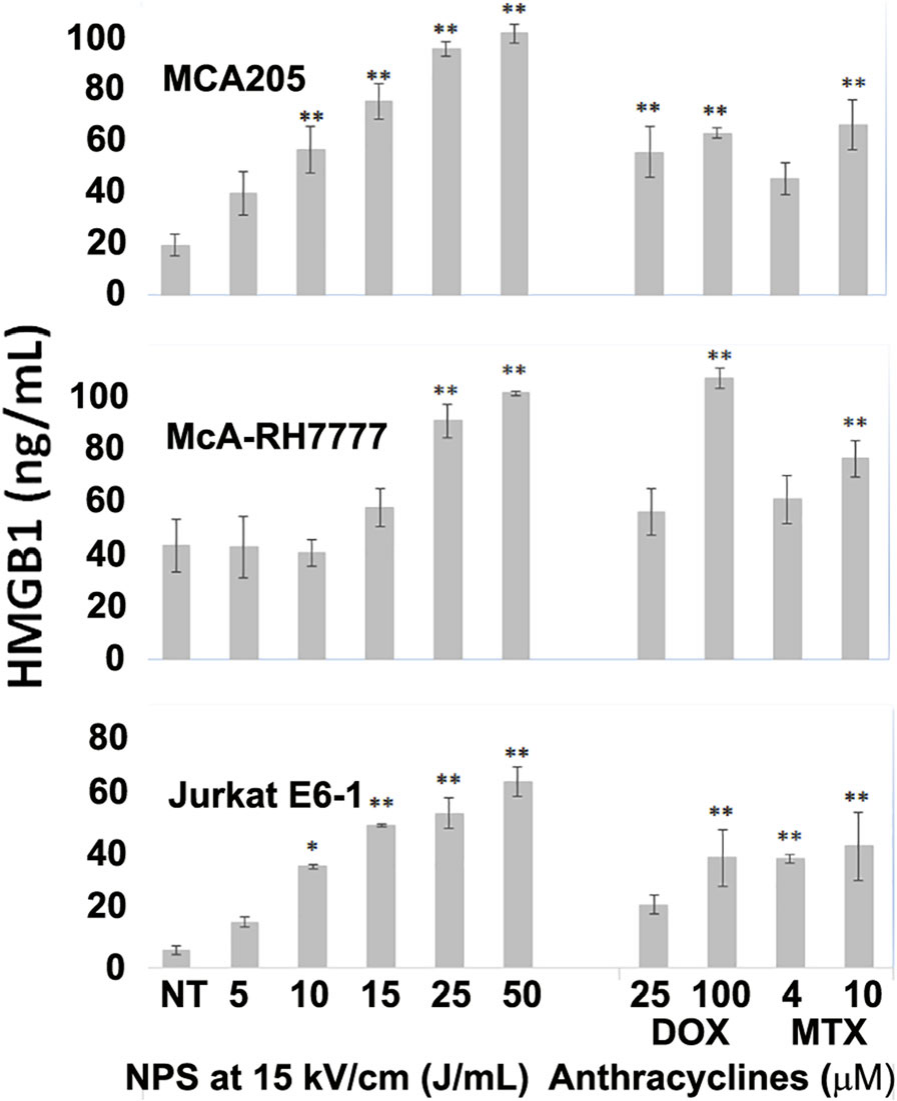
HMGB1 released by three cell lines 24 h after treatment with either NPS, or DOX or MTX. The number of pulses applied to achieve the indicated J/mL for all cell lines are indicated above the MCA205 plot. Significant difference from untreated controls by one-way ANOVA with between group analysis performed using the Dunnett’s test. *p < 0.05; **p < 0.01

The levels of HMGB1 24 h post-NPS were energy-dependent and, similar to the expression of ecto-CRT, continued to increase as the treatment energy increased for all of the three cell lines. HMGB1 concentrations after NPS treatment reached or exceeded those measured after anthracycline treatment once energies reached between 10 and 25 J/mL (Fig. 6).

## Discussion

NPS is an effective, non-thermal therapy that can eliminate tumors without recurrence [25, 28–31]. In our previously published work, we demonstrated a CD8-dependent immune response after NPS treatment (400 pulses, 100 ns, 50 kV/cm) of primary, isotopic McA-RH7777 liver tumors [9]. Partial or total inhibition of secondary tumor growth was observed after rats were given a secondary challenge of McA-RH7777 cells. The ER-stress response caused by the creation of nanopores and subsequent ATP release, as well as the ability to induce an adaptive immune response, lead us to suspect that the cell death modality of NPS was immunogenic cell death (ICD). In this study we demonstrate that when NPS is delivered at specific energies, it can induce apoptotic cell death as well as the emission of three key markers of ICD.

### Electrical properties of NPS and cell viability

Cell viability is dependent on energy delivery and current flow across the cells. The ED_50_ range of ablative efficacy is 10–15 J/mL for all field strengths above 10 kV/cm when the current flow across the cells is over 50A/cm^2^. When the current density is reduced to zero by increasing medium resistivity, cell viability increases dramatically to the point where no cells die after exposure to 16 J/mL. This increased viability in low conductivity media is somewhat controversial since some previous reports have observed that for low pulse numbers, the cell viability decreases in low conductivity media [26, 27] Possible explanations of these contradictory observations include: 1) Ionic dependence of membrane charging requirements to generate nanopores; 2) Maxwell stress tensor inducing a stretching force on the membrane that increases in low conductivity or, simply 3) The dependence of membrane properties on medium conductivity [13]. The fact is that these low conductivity solutions are definitely not physiological and can have many effects on cells. We can only conclude that NPS-induced cell death requires multiple pulses of current flow through cells.

### Evidence of apoptosis after NPS treatment

Several studies generated in other laboratories found that NPS triggers apoptosis in a variety of cell lines [8, 32–34]. We sought to independently verify this occurrence in our own laboratory by assaying the levels of activated caspases 3/7 as well as measuring the percentage of cells undergoing the stages of apoptotic cell death through the presence of cell surface phosphatidylserine (PS). Caspases 3/7 are effector caspases involved in initiating the cascade of cellular death-related events in caspase-3 dependent apoptosis. One of the events following initiation of apoptosis is the translocation of PS to the outer leaflet of the cell membrane, making both PS translocation and caspase-3 activation reliable markers of the process. Rather than increasing linearly with energy, we observe peak caspase activation in the range of 10–20 J/mL followed by a drop off around 25 J/mL. The magnitude of this peak varies considerably among cell lines in the range between 40% to 800%. Similarly, at 3 h the percent of cells undergoing the early stages of apoptosis drops off at 25 J/mL in both the MCA205 and McA-RH7777 cell lines. The Jurkat E6-1 cell line differed in that the percent of cells undergoing early apoptosis continued to increase with energy. It’s important to note, however, that the number of total cells at energies at or above 25 J/mL is significantly less than at lower energies. This is supported by cell viability data indicating that when cells were exposed to energies above 20 J/mL cell viability fell to 5% or less (Fig. 1). Since the total number of cells releasing activated caspase is much smaller at higher energies, this could account for decreased levels of total activated caspase at energies above 20 J/mL, even if the percent of cells undergoing apoptosis increases. Overall, higher energy treatments appear to cause a large number of cells to succumb immediately. Those that remain are typically at much later stages of cell death even by 3 h post-treatment. This is past the point of viability when we would expect to see activated caspase expression.

### Caspase-3 dependent apoptosis and immunogenic cell death

Caspase-3 is also important because of its role in the signaling cascade that leads to immunogenic cell death. When cells were genetically engineered to contain a caspase-3 “death switch” [35], its activation through the administration of doxorubicin lead to the expression of some DAMPs (HMGB1, HSP90) in vitro and regression of tumors in vivo. However, expression of CRT on the surface of these cells was not observed. This may be due to the direct activation of caspase-3 with the death switch which circumvents the need for caspase-8, an upstream initiator caspase. Caspase-8 activation initiates the caspase-3 cascade and also mediates the BAP31-dependent activation of BAX/BAK proteins leading to ER-stress and ecto-CRT expression [36]. Activation of the caspase-3 pathway alone may trigger the emission of some DAMPs, however upstream activation of the initiator caspase-8 may be required for the translocation of calreticulin to the cell surface, particularly in Type I ICD where certain cell death pathways need to be activated to trigger ER-stress.

Doxorubicin, an anthracycline and a Type I ICD inducer, is dependent on caspase-8 activation and stimulation of the death pathway to stimulate ER stress for its immunogenic properties [37]. Type II ICD inducers such as Hypericin photodynamic therapy (Hyp-PDT) directly stimulate ER stress in a manner that is independent of the caspase-8 pathway [38, 39]. Similar to Hyp-PDT, NPS triggers direct physical stress to the ER and to the mitochondria. Therefore it is most likely inducing cell death through one of the intrinsic programmed death pathways that don’t rely on caspase-8 activation [38, 39]. Caspase-dependent apoptosis may be initiated by direct ER-stress through the caspase-12 pathway or from mitochondrial stress and the release of cytochrome c through caspase-9. All three caspase-dependent pathways converge at the activation of caspase-3. There is an additional programmed cell death pathway that is caspase-independent and triggered directly by mitochondrial apoptotic proteins [39]. More direct investigation into which of the actual apoptotic pathways NPS initiates will be needed before we can draw any conclusions.

### Expression of ecto-CRT

As noted above the induction of ecto-CRT expression is dependent on either direct or indirect ER-stress and ROS production [10]. Once CRT has been translocated to the outer leaflet of the cell membrane it serves as an “eat me” signal by binding specifically to the LDL-receptor-related protein 1 (LRP1; alternate name CD91) on the surface of dendritic cells (DC), and initiating the clearance of dead cell material through phagocytosis. CRT exposure has been shown to dictate the immunogenicity of cancer cell death [40].

In our previous study, we demonstrated that NPS treatment led to ecto-CRT appearance within 2 h in three different cancer cell lines, including McA-RH7777 cells, using immunofluorescence microscopy [9]. In our current study we measured the percent of cells that expressed ecto-CRT in the McA-RH7777 as well as the MCA205 and Jurkat E6-1 cell lines using flow cytometry. We also co-labeled for cell viability with Zombie Aqua to determine the percentage of viable cells expressing ecto-CRT 24 h after treatment. The percent of cells expressing cell surface CRT was comparable to those of the anthracyclines at energies at or above 25 J/mL. It is interesting to note that, although the percent of cells expressing cell surface CRT continues to increase with energy, the percent that are viable drastically decreases after 15 J/mL (Fig. 4a). There is also a sharp decline in the total number of cells left remaining at 24 h when treatment energies are greater than 15 J/mL (Fig. 4b). This is in accordance with cell viability data demonstrating that less than 5% of cells are viable after being treated with energies greater than 15 J/mL.

It appears that when cultured cells are treated with energies at or above 25 J/mL a large number succumb to NPS immediately, leaving a small number of remaining cells that are typically at the late stages of cell death. This large amount of cell death is likely due to heavy cellular damage at higher energies caused when pores form in the cell membrane. These pores allow small solutes to diffuse across the membrane while larger solutes remain inside. This causes an osmotic gradient that attracts water and induces swelling and necrosis [41]. Cells that do not swell to the point of immediate death following NPS exposure are, nonetheless, affected by the physical stress imposed by the treatment and many may die at intervals that are much shorter than it would normally take them to complete the entire apoptotic process [42]. The flux of Ca^2+^ due to the temporary permeability of the cell and organelle membranes triggers direct ER-stress, and may cause calreticulin to be translocated to the surface of the cell very quickly. Hyp-PDT, another direct ER-stress inducer was observed to mobilize CRT to the cell surface within 30-min treatment, a much shorter time frame than that reported for anthracyclines [38, 40].

This ER-stress would likely trigger the initiation of apoptosis through the induction of downstream pathways, however, if the treatment caused enough damage to cells and organelles, they may reach a threshold at which they would be unable to repair themselves and instead die of necrosis [42–44]. One mechanism could involve the loss of ATP after NPS treatment. The permeabilization of the mitochondria caused by nanosecond pulses results in loss of membrane potential and depletion of cellular ATP [23, 45]. With enough pulse energy, the stress to the mitochondria could deplete enough ATP to cause immediate cell death [46]. This is one possible explanation for why a large number of cells are expressing cell surface CRT at higher energies, despite the fact that the majority are already typically at the latest stages of cell death only 3 h post-treatment. Additional studies will be carried out to characterize the mechanisms deciding cell fate after NPS treatment and their specific triggers.

### ATP secretion

ATP acts as a “find me” signal for dendritic cells (DC) and influences their maturation and secretion of IL-1β [47]. IL-1β is required for the polarization of CD8^+^ cytotoxic T-cells towards an anti-tumor response [48]. Secretion of ATP depends on a complex pathway involving caspase- and PANX1-dependent lysosomal exocytosis [49]. ATP is an early stage apoptotic marker and is secreted when cells are still viable. However, as noted above, too much depletion can lead quickly to necrosis. The ATP released from both MCA205 and McA-RH7777 cells was bell shaped with a sharp peak at 15 J/mL (Fig. 5) followed by a drop-off at 25 J/mL. The ATP release was significantly higher in MCA205 cells at 15 J/mL than that in untreated cells and was higher than all concentrations of both doxorubicin and mitoxantrone. Since cells that are in later stages of cell death are not actively secreting ATP at 24 h, the drop-off at 25 J/mL is likely due to the sharp decline in the number of viable cells at higher energies, as non-viable cells have already been depleted of ATP. Jurkat E6-1 cells did not secrete high levels of ATP whether treated with NPS or with an anthracycline, suggesting these cells may simply be lower expressors of this marker.

### HMGB1 release

HMGB1 is a late stage DAMP that is passively released when cells are undergoing secondary necrosis [50]. It binds to TLR2, TLR4 and RAGE on the surface of immune cells and has potent leukocyte chemotactic activity by forming a complex with C-X-C chemokine ligand 12 (CXCL12) and signaling through C-X-C receptor 4. It also assists in the maturation and migration of DCs into lymphoid tissues [51]. Levels of HMGB1 increased as treatment energy increased and reached or exceeded those in the anthracycline treated groups between 10 and 25 J/mL, 24 h after treatment (Fig. 6). The levels of HMGB1 release increase substantially after 15 J/mL, most likely due to the large number cells that are undergoing secondary necrosis at these higher energies.

### NPS-induced cell death is energy dependent

NPS cell death properties, including the expression levels of different DAMPs, are highly dependent on energy delivery. We know there is a directional shift in how the cells respond to treatment energies over 15–20 J/mL. The measured caspase activation and ATP secretion tend to increase with increasing energy up until 15–20 J/mL when levels drop off. The number of viable cells also drops below 5% at energies higher than 20 J/mL. Since these are markers of early events in the programmed cell death process, we believe the drop off is primarily due to the larger ratio of cells that succumb quickly after NPS treatment at higher energies, leaving few viable cells available to express these markers. Additionally, the few cells that remain at energies at or above 25 J/mL are mostly in the later stages of cell death, having already been depleted of ATP. This is reflected by HMGB1, a DAMP released during the late stages of secondary necrosis that is expressed at the highest levels at energies at or above 25 J/mL. The percentage of cells that express ecto-CRT (the third key DAMP) continues to rise with increasing energy and spikes at 25 J/mL even as cell viability and the total cell number plummets. We speculate that direct ER-stress may initiate apoptosis and the translocation of CRT to the membrane, but be cut short if the amount of cellular damage is beyond repair, recommitting the cell to a necrotic fate. If CRT is expressed early in the cell death process, it may still function as an “eat me” signal, even if the cell is quickly recommitted to a necrotic fate.

It is likely that cell death triggered by NPS exists in a continuum between apoptosis and necrosis and that the ultimate fate of each cell is dependent on the energy “dose” received and the resulting level of physical damage to the cell. Given its ability to induce stress to both the ER and the mitochondria it seems likely that NPS is a Type II inducer of ICD. Nevertheless, its novel properties require more characterization before we can fully classify the cell death pathways involved and how they mediate the expression of DAMPS and their temporal presentation during the cell death process.

Recognition of abnormal cancer antigens hinges on the ability of DAMPs to traffic DCs and other leukocytes to the site of the tumor in order to stimulate a robust immune response. We know from the literature that each of the 3-key DAMPs has its own unique role in stimulating an immune response, however, we see a different expression profile for each of these DAMPs that is dependent upon treatment energy parameters and unique to NPS. It is clear that some markers of apoptosis and ICD are expressed at the highest levels at lower energies (caspase-3/ATP:10–20 J/mL) while others continue to increase in expression as energy increases (CRT/HMGB1: 25–50 J/mL). This is likely related to the type of cell death and the respective ratios of these modalities at different NPS treatment energies.

We are still in the process of learning how the DAMP expression signature influences the immunogenicity of NPS treatments and how this translates to response in vivo. Previously, we demonstrated that NPS treatment is able to show an inhibitory effect on secondary tumor growth. Now we need to ascertain how varying treatment energy will influence the efficacy of this effect. Our future endeavors will consist of characterizing the types of cell death and associated pathways after NPS treatment at different energies to discern how these give rise to the expression of DAMPS and the recruitment of immune cells in vivo. This will help us determine the optimal treatment energy parameters needed to induce a cell death signature that will stimulate the most robust adaptive immune response.

## Conclusions

We conclude that NPS is a physical modality that is able to induce expression of the three consensus DAMPS indicative of immunogenic cell death. The NPS energy required using 100 ns pulses at 15 kV/cm is 10–20 J/mL and a minimum of 50 A/cm^2^ to cells in vitro. Further studies are needed to determine the optimal pulse parameters and energy to produce the most robust adaptive immune response in solid tumors.

## Abbreviations

CRT: Calreticulin
DAMP: Danger associated molecular pattern
DC: Dendritic cell
DOX: Doxorubicin
ecto-CRT: Calreticulin on cell surface
HMGB1: High mobility group box 1
ICD: Immungenic cell death
MTX: Mitoxantrone
NPS: Nano-Pulse Stimulation
ROS: Reactive oxygen species
TME: Tumor microenvironment
T_reg_: Regulatory T cells

## References

[1] Schoenbach KH, Pakhomov AG, Miklavcic D, Markov MS (2010). Bioelectric effect of intense nanosecond pulses. Advanced Electroporation Techniques in Biology and Medicine.

[2] Pakhomov AG, Gianulis E, Vernier PT, Semenov I, Xiao S, Pakhomova ON (2015). Multiple nanosecond electric pulses increase the number but not the size of long-lived nanopores in the cell membrane. Biochim Biophys Acta.

[3] Vernier PT, Sun Y, Marcu L, Salemi S, Craft CM, Gundersen MA (2003). Calcium bursts induced by nanosecond electric pulses. Biochem Biophys Res Commun.

[4] White JA, Blackmore PF, Schoenbach KH, Beebe SJ (2004). Stimulation of capacitative calcium entry in HL-60 cells by nanosecond pulsed electric fields. J Biol Chem.

[5] Pakhomova ON, Khorokhorina VA, Bowman AM, Rodaite-Riseviciene R, Saulis G, Xiao S, Pakhomov AG (2012). Oxidative effects of nanosecond pulsed electric field exposure in cells and cell-free media. Archives Biochem Biophys.

[6] Nuccitelli R, Lui K, Kreis M, Athos B, Nuccitelli P (2013). Nanosecond pulsed electric field stimulation of reactive oxygen species in human pancreatic cancer cells is Ca^2+^-dependent. Biochem Biophys Res Commun.

[7] Schoenbach KH, Beebe SJ, Buescher ES (2001). Intracellular effect of ultrashort electrical pulses. Bioelectromagnetics.

[8] Chen R, Sain NM, Harlow KT, Chen YJ, Shires PK, Heller R, Beebe SJ (2014). A protective effect after clearance of orthotopic rat hepatocellular carcinoma by nanosecond pulsed electric fields. Eur J Cancer.

[9] Nuccitelli R, Berridge JC, Mallon Z, Kreis M, Athos B, Nuccitelli P (2015). Nanoelectroablation of murine tumors triggers a CD8-dependent inhibition of secondary tumor growth. PLoS One.

[10] Kroemer G, Galluzzi L, Kepp O, Zitvogel L (2013). Immunogenic cell death in cancer therapy. Annu Rev Immunol.

[11] Galluzzi L, Maiuri MC, Vitale I, Zischka H, Castedo M, Zitvogel L, Kroemer G (2007). Cell death modalities: classification and pathophysiological implications. Cell Death Differ.

[12] Hanahan D, Weinberg RA (2011). Hallmarks of cancer: the next generation. Cell.

[13] Adkins I, Fucikova J, Garg AD, Agostinis P, Spisek R (2014). Physical modalities inducing immunogenic tumor cell death for cancer immunotherapy. Oncoimmunology.

[14] Krysko DV, Garg AD, Kaczmarek A, Krysko O, Agostinis P, Vandenabeele P (2012). Immunogenic cell death and DAMPs in cancer therapy. Nat Rev Cancer.

[15] Fucikova J, Kralikova P, Fialova A, Brtnicky T, Rob L, Bartunkova J, Spisek R (2011). Human tumor cells killed by anthracyclines induce a tumor-specific immune response. Cancer Res.

[16] Vacchelli E, Senovilla L, Eggermont A, Fridman WH, Galon J, Zitvogel L, Kroemer G, Galluzzi L (2013). Trial watch: chemotherapy with immunogenic cell death inducers. Oncoimmunology.

[17] Vacchelli E, Aranda F, Eggermont A, Galon J, Sautes-Fridman C, Cremer I, Zitvogel L, Kroemer G, Galluzzi L (2014). Trial watch: chemotherapy with immunogenic cell death inducers. Oncoimmunology.

[18] Garg AD, Galluzzi L, Apetoh L, Baert T, Birge RB, Bravo-San Pedro JM, Breckpot K, Brough D, Chaurio R, Cirone M (2015). Molecular and translational classifications of DAMPs in immunogenic cell death. Front Immunol.

[19] Workenhe ST, Mossman KL (2014). Oncolytic virotherapy and immunogenic cancer cell death: sharpening the sword for improved cancer treatment strategies. Mol Ther.

[20] Garg AD, Krysko DV, Vandenabeele P, Agostinis P (2012). The emergence of phox-ER stress induced immunogenic apoptosis. Oncoimmunology.

[21] Dudek AM, Garg AD, Krysko DV, De RD, Agostinis P (2013). Inducers of immunogenic cancer cell death. Cytokine Growth Factor Rev.

[22] Kepp O, Senovilla L, Vitale I, Vacchelli E, Adjemian S, Agostinis P, Apetoh L, Aranda F, Barnaba V, Bloy N (2014). Consensus guidelines for the detection of immunogenic cell death. Oncoimmunology.

[23] Batista NT, Wu YH, Gundersen MA, Miklavcic D, Vernier PT (2012). Nanosecond electric pulses cause mitochondrial membrane permeabilization in Jurkat cells. Bioelectromagnetics.

[24] Carr-Brendel V, Markovic D, Smith M, Taylor-Papadimitriou J, Cohen EP (1999). Immunity to breast cancer in mice immunized with X-irradiated breast cancer cells modified to secrete IL-12. Journal of immunotherapy (Hagerstown, Md : 1997).

[25] Nuccitelli R, Pliquett U, Chen X, Ford W, James SR, Beebe SJ, Kolb JF, Schoenbach KH (2006). Nanosecond pulsed electric fields cause melanomas to self-destruct. Biochem Biophys Res Commun.

[26] Silve A, Leray I, Leguebe M, Poignard C, Mir LM (2015). Cell membrane permeabilization by 12-ns electric pulses: Not a purely dielectric, but a charge-dependent phenomenon. Bioelectrochemistry.

[27] Silve A, Leray I, Poignard C, Mir LM (2016). Impact of external medium conductivity on cell membrane electropermeabilization by microsecond and nanosecond electric pulses. Sci Rep.

[28] Nuccitelli R, Tran K, Athos B, Kreis M, Nuccitelli P, Chang KS, Epstein EH, Tang JY (2012). Nanoelectroablation therapy for murine basal cell carcinoma. Biochem Biophys Res Commun.

[29] Nuccitelli R, Tran K, Lui K, Huynh J, Athos B, Kreis M, Nuccitelli P, De Fabo EC (2012). Non-thermal Nanoelectroablation of UV-Induced Murine Melanomas Stimulates an Immune Response. Pigment Cell Melanoma Res.

[30] Nuccitelli R, Chen X, Pakhomov AG, Baldwin WH, Sheikh S, Pomicter JL, Ren W, Osgood C, Swanson RJ, Kolb JF (2009). A new pulsed electric field therapy for melanoma disrupts the tumor's blood supply and causes complete remission without recurrence. Int J Cancer.

[31] Nuccitelli R, Huynh J, Lui K, Wood R, Kreis M, Athos B, Nuccitelli P (2013). Nanoelectroablation of human pancreatic carcinoma in a murine xenograft model without recurrence. Int J Cancer.

[32] Beebe SJ, Fox P, Rec LJ, Somers K, Stark RH, Schoenbach KH (2002). Nanosecond pulsed electric field (nsPEF) effects on cells and tissues: Apoptosis induction and tumor growth inhibition. IEEE Transactions on Plasma Science.

[33] Beebe SJ, Fox PM, Rec LJ, Willis EL, Schoenbach KH (2003). Nanosecond, high-intensity pulsed electric fields induce apoptosis in human cells. FASEB J.

[34] Hall EH, Schoenbach KH, Beebe SJ (2007). Nanosecond pulsed electric fields induce apoptosis in p53-wildtype and p53-null HCT116 colon carcinoma cells. Apoptosis.

[35] Melis MH, Simpson KL, Dovedi SJ, Welman A, MacFarlane M, Dive C, Honeychurch J, Illidge TM (2013). Sustained tumour eradication after induced caspase-3 activation and synchronous tumour apoptosis requires an intact host immune response. Cell Death Differ.

[36] Panaretakis T, Kepp O, Brockmeier U, Tesniere A, Bjorklund AC, Chapman DC, Durchschlag M, Joza N, Pierron G, van Endert P (2009). Mechanisms of pre-apoptotic calreticulin exposure in immunogenic cell death. EMBO J.

[37] Casares N, Pequignot MO, Tesniere A, Ghiringhelli F, Roux S, Chaput N, Schmitt E, Hamai A, Hervas-Stubbs S, Obeid M (2005). Caspase-dependent immunogenicity of doxorubicin-induced tumor cell death. J Exp Med.

[38] Garg AD, Krysko DV, Vandenabeele P, Agostinis P (2012). Hypericin-based photodynamic therapy induces surface exposure of damage-associated molecular patterns like HSP70 and calreticulin. Cancer Immunol Immunother.

[39] Panzarini E, Inguscio V, Dini L (2013). Immunogenic cell death: can it be exploited in PhotoDynamic Therapy for cancer?. Biomed Res Int.

[40] Obeid M, Tesniere A, Ghiringhelli F, Fimia GM, Apetoh L, Perfettini JL, Castedo M, Mignot G, Panaretakis T, Casares N (2007). Calreticulin exposure dictates the immunogenicity of cancer cell death. Nat Med.

[41] Pakhomova ON, Gregory BW, Semenov I, Pakhomov AG (2013). Two modes of cell death caused by exposure to nanosecond pulsed electric field. PLoS One.

[42] Ibey BL, Pakhomov AG, Gregory BW, Khorokhorina VA, Roth CC, Rassokhin MA, Bernhard JA, Wilmink GJ, Pakhomova ON (2010). Selective cytotoxicity of intense nanosecond-duration electric pulses in mammalian cells. Biochim Biophys Acta.

[43] Draeger A, Schoenauer R, Atanassoff AP, Wolfmeier H, Babiychuk EB (2014). Dealing with damage: plasma membrane repair mechanisms. Biochimie.

[44] Thompson GL, Roth CC, Dalzell DR, Kuipers M, Ibey BL (2014). Calcium influx affects intracellular transport and membrane repair following nanosecond pulsed electric field exposure. J Biomed Opt.

[45] Borutaite V (2010). Mitochondria as decision-makers in cell death. Environ Mol Mutagen.

[46] Leist M, Single B, Castoldi AF, Kuhnle S, Nicotera P (1997). Intracellular adenosine triphosphate (ATP) concentration: a switch in the decision between apoptosis and necrosis. J Exp Med.

[47] Elliott MR, Chekeni FB, Trampont PC, Lazarowski ER, Kadl A, Walk SF, Park D, Woodson RI, Ostankovich M, Sharma P (2009). Nucleotides released by apoptotic cells act as a find-me signal to promote phagocytic clearance. Nature.

[48] Ghiringhelli F, Apetoh L, Tesniere A, Aymeric L, Ma Y, Ortiz C, Vermaelen K, Panaretakis T, Mignot G, Ullrich E (2009). Activation of the NLRP3 inflammasome in dendritic cells induces IL-1beta-dependent adaptive immunity against tumors. Nat Med.

[49] Martins I, Wang Y, Michaud M, Ma Y, Sukkurwala AQ, Shen S, Kepp O, Metivier D, Galluzzi L, Perfettini JL (2014). Molecular mechanisms of ATP secretion during immunogenic cell death. Cell Death Differ.

[50] Apetoh L, Ghiringhelli F, Tesniere A, Obeid M, Ortiz C, Criollo A, Mignot G, Maiuri MC, Ullrich E, Saulnier P (2007). Toll-like receptor 4-dependent contribution of the immune system to anticancer chemotherapy and radiotherapy. Nat Med.

[51] Manfredi AA, Capobianco A, Esposito A, De Cobelli F, Canu T, Monno A, Raucci A, Sanvito F, Doglioni C, Nawroth PP (2008). Maturing dendritic cells depend on RAGE for in vivo homing to lymph nodes. J Immunol.

